# Importance of TLR2 on Hepatic Immune and Non-Immune Cells to Attenuate the Strong Inflammatory Liver Response During *Trypanosoma cruzi* Acute Infection

**DOI:** 10.1371/journal.pntd.0000863

**Published:** 2010-11-02

**Authors:** Eugenio Antonio Carrera-Silva, Natalia Guiñazu, Andrea Pellegrini, Roxana Carolina Cano, Alfredo Arocena, Maria Pilar Aoki, Susana Gea

**Affiliations:** Inmunología, Departamento de Bioquímica Clínica, Facultad de Ciencias Químicas, CIBICI-CONICET, Universidad Nacional de Córdoba, Córdoba, Argentina; Institut Pasteur, France

## Abstract

**Background:**

Toll-like receptors (TLR) and cytokines play a central role in the pathogen clearance as well as in pathological processes. Recently, we reported that TLR2, TLR4 and TLR9 are differentially modulated in injured livers from BALB/c and C57BL/6 (B6) mice during *Trypanosoma cruzi* infection. However, the molecular and cellular mechanisms involved in local immune response remain unclear.

**Methodology/Principal Findings:**

In this study, we demonstrate that hepatic leukocytes from infected B6 mice produced higher amounts of pro-inflammatory cytokines than BALB/c mice, whereas IL10 and TGFβ were only released by hepatic leukocytes from BALB/c. Strikingly, a higher expression of TLR2 and TLR4 was observed in hepatocytes of infected BALB/c mice. However, in infected B6 mice, the strong pro-inflammatory response was associated with a high and sustained expression of TLR9 and iNOS in leukocytes and hepatic tissue respectively. Additionally, co-expression of gp91- and p47-phox NADPH oxidase subunits were detected in liver tissue of infected B6 mice. Notably, the pre-treatment previous to infection with Pam3CSK4, TLR2-agonist, induced a significant reduction of transaminase activity levels and inflammatory foci number in livers of infected B6 mice. Moreover, lower pro-inflammatory cytokines and increased TGFβ levels were detected in purified hepatic leukocytes from TLR2-agonist pre-treated B6 mice.

**Conclusions/Significance:**

Our results describe some of the main injurious signals involved in liver immune response during the *T. cruzi* acute infection. Additionally we show that the administration of Pam3CSk4, previous to infection, can attenuate the exacerbated inflammatory response of livers in B6 mice. These results could be useful to understand and design novel immune strategies in controlling liver pathologies.

## Author Summary

*Trypanosoma cruzi*, an obligate intracellular protozoan, is the etiological agent of Chagas Disease that represents an important public health burden in Latin America. The infection with this parasite can lead to severe complications in cardiac, liver and gastrointestinal tissue depending on the strain of parasite and host genetics. Recently, we reported a fatal liver injury in *T. cruzi* infected B6 mice. However, the local immune response against this parasite is poorly understood. This work highlights some of the molecular and cellular mechanisms involved in liver pathology during the acute phase of infection. Using two mouse strains with different genetic backgrounds and responses to infection, B6 and BALB/c, we found that infected B6 mice develop a strong pro-inflammatory environment associated with high TLR9 expression. Conversely, infected BALB/c mice showed a more balanced inflammatory response in liver. Moreover, higher TLR2 and TLR4 expression were found only in hepatocytes from BALB/c. These data emphasize the importance of an adequate integration of signalling between immune and non-immune cells to define the outcome of infection. In addition, the pre-treatment with TLR2-agonist reverts the strong pro-inflammatory environment in *T. cruzi* infected B6 mice. These results could be useful in the understanding and design of novel immune strategies in controlling liver pathologies.

## Introduction

Accumulative evidences demonstrated that the liver has specific immunological properties and contains a large number of resident and non-resident cells that participate in the regulation of inflammatory and immune responses [1], [2].

Kupffer cells are among the first cells that orchestrate the inflammatory response under many pathological conditions and they produce pro-inflammatory cytokines and several chemokines after pathogen stimulation. Interestingly, while TNFα and IL6 released by Kupffer cells are involved in hepatic inflammation and liver cell death, paradoxically they also mediate regeneration of the liver after injury [1], [3]. Importantly, hepatic infiltration of neutrophils participates in early response to cellular stress, and their activation is critical for host defence but can also cause additional tissue damage. Thus, proteases and reactive oxygen species (ROS) released by neutrophils can result in mitochondrial dysfunction and eventually in necrotic cell death [4]. Hepatocytes are the most abundant cells in the liver, and it has been shown that they have an important role not only in detoxification but also in controlling systemic innate immunity via production of secreted PRRs and complement components, while also acting as antigen presenting cells [2], [3]. Primary culture of hepatocytes express mRNA for all TLRs and respond to TLR2 and TLR4 ligands [1]. Recently, it has also been reported that hepatocytes are desensitized by LPS in a TLR4 signalling-dependent manner [5]. However, LPS response is mediated by several hepatic cell populations, which are part of a cellular network involved in the hepatic wound healing and regenerative response [1], [6], [7].

Although, the liver is the target of a wide range of microbes including *Listeria*, *Salmonella* and *Plasmodium species*, there are few data related to the implication of *Trypanosoma cruzi* experimental infection in liver and the relevance of the innate immune response in this organ [8], [9].

The *T. cruzi* parasite, an obligate intracellular protozoan, is the causative agent of Chagas disease and represents an important public health burden in Latin America. Nowadays, this infection affects at 9 million people, and more than 30,000 new cases occur every year (WHO; 2007. http://www.who.int/mediacentre/news/notes/2007/np16/en/index.html). Chagas disease is characterized by two distinct phases, the acute phase which lasts 2–4 months, involves a number of parasites detected in the blood stream as well as in host tissues followed by a life-long chronic phase. Parasite persistence eventually leads to severe complications in cardiac and gastrointestinal tissue. However, *T cruzi* also infects the reticuloendothelial system including the liver, spleen and bone marrow. Hepatic affection during acute infection by *T. cruzi* has been demonstrated both in humans and experimental animals [10]–[12]. Despite extensive experimental investigations, the proper mechanism developed by the host to limit acute *T. cruzi* infection has not been fully elucidated. We have recently reported severe hepatic injury in B6 mice infected with *T. cruzi*, Tulahuén strain. The high mortality in B6 mice was associated with an unbalanced pro-inflammatory cytokine profile, a decreased TLR2 and TLR4 and an increased TLR9 expression in the liver compared to infected BALB/c mice [13]. In this study, we hypothesized that the injurious and non-controlled local inflammatory response in the liver depends on the adequate activation of immuno-modulatories signalling, such as TLR2 and TGFβ cytokine, on both non-immune and immune liver cells during *T. cruzi* infection. With the purpose to gain more knowledge about the mechanisms involved in hepatic injury during *T. cruzi* infection, we analyzed the ability of hepatic leukocytes to produce cytokines and inflammatory mediators, such as NO and ROS, which could directly and/or indirectly trigger tissue injury. In parallel, we also investigated liver iNOS and nicotinamide adenine dinucleotide phosphate (NAPDH) oxidase gp91 and p47 phox expression, the main inducers of NO and ROS, respectively. In addition, we evaluated the induction of TLR2, TLR4 and TLR9 in leukocytes infiltrating the liver and hepatocytes during *T. cruzi* acute infection. Taking into account that the use of TLR-agonists is a potential therapeutic approach and a promissing strategy to treat infectious diseases [14]–[16], we also evaluated if Pam3CSK4 (TLR2-TLR1 agonist) pre-treatment before challenge with *T. cruzi* is able to modulate the strong liver inflammatory response elicited in B6 mice. Our results show for the first time that, high and sustained levels of NO, ROS and pro-inflammatory cytokines would be dangerous signals to the liver during *T, cruzi* acute infection. The administration of Pam3CSk4, previous to infection, clearly attenuated the exacerbated inflammation in liver of B6 mice during acute *T. cruzi* infection, Tulahuén strain.

## Materials and Methods

### Mice, infection and parasitemia

BALB/c and B6 mice were purchased from the Faculty of Veterinary Sciences (National University of La Plata, Bs As, Argentina) and maintained according to the National Research Council's guide for the care and use of laboratory animals. Protocols were approved by the Animals Experimentation Ethics Committee, Faculty of Chemical Science, National University of Cordoba. Six to eight week-old females of both mouse strains were infected intraperitoneally with 5×10^3^ *T. cruzi* blood-derived trypomastigotes, Tulahuén strain. Non-infected mice for each strain were used as control. Parasites were maintained by serial passages from mouse to mouse.

### TLR-agonist pre-treatment

Six to eight week-old B6 mice were pre-treated 24hs before to infection with 10 ug of Pam3CSK4. At the next day the mice were intraperitoneally infected with 5000 parasites. In addition, non-infected mice were challenged with the same doses of TLR2-ligand and controlled along the acute phase, 24 days post infection (dpi). The different groups consist of infected mice, pre-treated/infected mice, pre-treated/non-infected mice and non-infected mice.

### Transaminase activity

Plasma glutamic-oxaloacetic transaminase (GOT) and glutamic-pyruvic transaminase (GPT) activities were measured as tissue damage index, employing a commercial kit (Wiener Lab) and performed according to appended protocol.

### Immunofluoroscence

Liver specimens were fixed in 4% paraformaldehyde and embedded in paraffin. Sections deparaffinized and rehydrated were permeabilized, blocked using Fc block, and incubated with primary antibody, rabbit polyclonal anti-TLR2 at 1/50, rabbit polyclonal anti-p47-phox at 1/200, goat anti-mouse TLR4 at 1/100, goat polyclonal anti-gp91at 1/400, or monoclonal anti-mouse TLR9 1/100 dilutions (Santa Cruz Biotechnology) in PBS containing 2% (w/v) BSA. After washing, the sections were incubated with FITC-conjugated anti-rabbit (1/200), TRITC-conjugated anti-goat (1/200), and FITC-conjugated anti-mouse (1/100). Nuclei were examined using DNA-binding fluorochome Hoechst 33258 (2 ug/ml). Slides were observed with a NIKON ECLIPSE TE-2000 U Microscope equipped with UV and fluorescence broadband.

### Flow cytometry analysis of liver leukocytes

Hepatic leukocytes were isolated from both infected and non-infected mouse strains as previously described [13]. Briefly, liver in RMPI medium containing 100 U/ml heparin plus 2% of FBS was passed through 100-µm nylon meshes; red blood cells were removed using lysis buffer (SIGMA) and finally hepatocytes were separated from leukocytes using isotonic 37% Percoll gradient (SIGMA). For flow cytometry, 1×10^6^ cells were stained using a standard protocol and the following Abs were used: FITC-labeled rat anti-mouse Ly-6G-Gr1 and F4/80 (eBioscience), FITC-labeled hamster anti-mouse CD3e mAb (BD Pharmingen), PE-labeled rat anti-mouse TLR2 and TLR4 and biotin anti-mouse TLR9 mAb (eBioscience). Stained samples were acquired using the Ortho Diagnostics System (Johnson and Johnson Company) flow cytometer, and data were recorded and analyzed using FlowJo software 5.7.2 (Tree Star, Inc.).

### RT-PCR assays

RNA was isolated from 50 mg of hepatic tissue using TRIZOL reagent (Invitrogen) as previously described [13]. The cDNA obtained was amplified by PCR using specific primers for gp91 and p47-phox. As an endogenous control GAPDH primer was used. Each primer pair was tested to reach the appropriate condition of amplification. The primer sequence (sense and antisense) from 5′ to 3′: **gp91** (GAAGACTCTGTATGGACGGC and GCCTGTGTCATTGTGATTTCCT); **p47-phox** (GACCTGTCGGAGAAGGTGGT and CTTCACGGGCAGTCCCATGA) **GAPDH** (ACCACCATGGAGAAGGCCGG and CTCAGTGTAGCCCAAGATGC).

Relative expression levels of the transcripts were estimated by standardization with internal control of GAPDH gene, and evaluated by densitometry using the GELPRO 3.1 analysis program.

### Cytokine assays

Hepatic leukocytes (2×10^6^/ml) from infected (14 and 21dpi) and non-infected mice (purified as described above) were cultured with RPMI medium (Sigma) containing 10% FBS and 40 ug/ml gentamycin for 72 h. ELISA assay was performed as previously described [13] for IL1β, IL4, IL6, IL10, IL12, TGFβ, TNFα and IFNγ cytokines. Standard curves were generated using recombinant cytokine for each of them (eBioscience).

### Nitrite determination

Supernatants (100 µl) were mixed with Griess reagent (Sigma, USA) at a ratio of 1∶1 and incubated at room temperature for 15 min. The absorbance was measured at 540 nm using a microplate reader and nitrite concentration was calculated in µM using a standard curve of sodium nitrite.

### Western blot assays

Livers were washed and lysed for 30 min on ice in lysis buffer (1% Triton X-100, 0.5% sodium deoxicholate, 9% SDS, 5% dithiothreitol (DTT), 1 mM sodium ortovanadate, 10 ug PMSF, 30 ug aprotinin in PBS). Aliquots of tissue lysates, diluted in SDS sample buffer, were separated on a 10% SDS-PAGE and western blot assays were performed as previously described [13]. The following antibodies were used: rabbit polyclonal anti-p47-phox at 1/200 dilution or goat polyclonal anti-gp91 (Santa Cruz Biotechnology) at 1/800 in PBS containing 1% (w/v) BSA. The assays were revealed using the ECL chemiluminescent system (Amersham Pharmacia Biotech) according to the manufacturer's instructions.

### Statistical analysis

Statistical significance among groups was assessed by ANOVA. To compare different experimental conditions an analysis of variance (Two-way or one-way ANOVA) with Bonferroni's post-hoc test was performed. A p-value <0.05 was considered significant.

## Results

### Hepatic leukocytes from B6 mice showed an exacerbated pro-inflammatory cytokine profile

During the acute phase of the inflammatory response, a variety of pro-inflammatory cytokines are released which can induce activation, danger or regenerative signalling.

**Figure 1:**
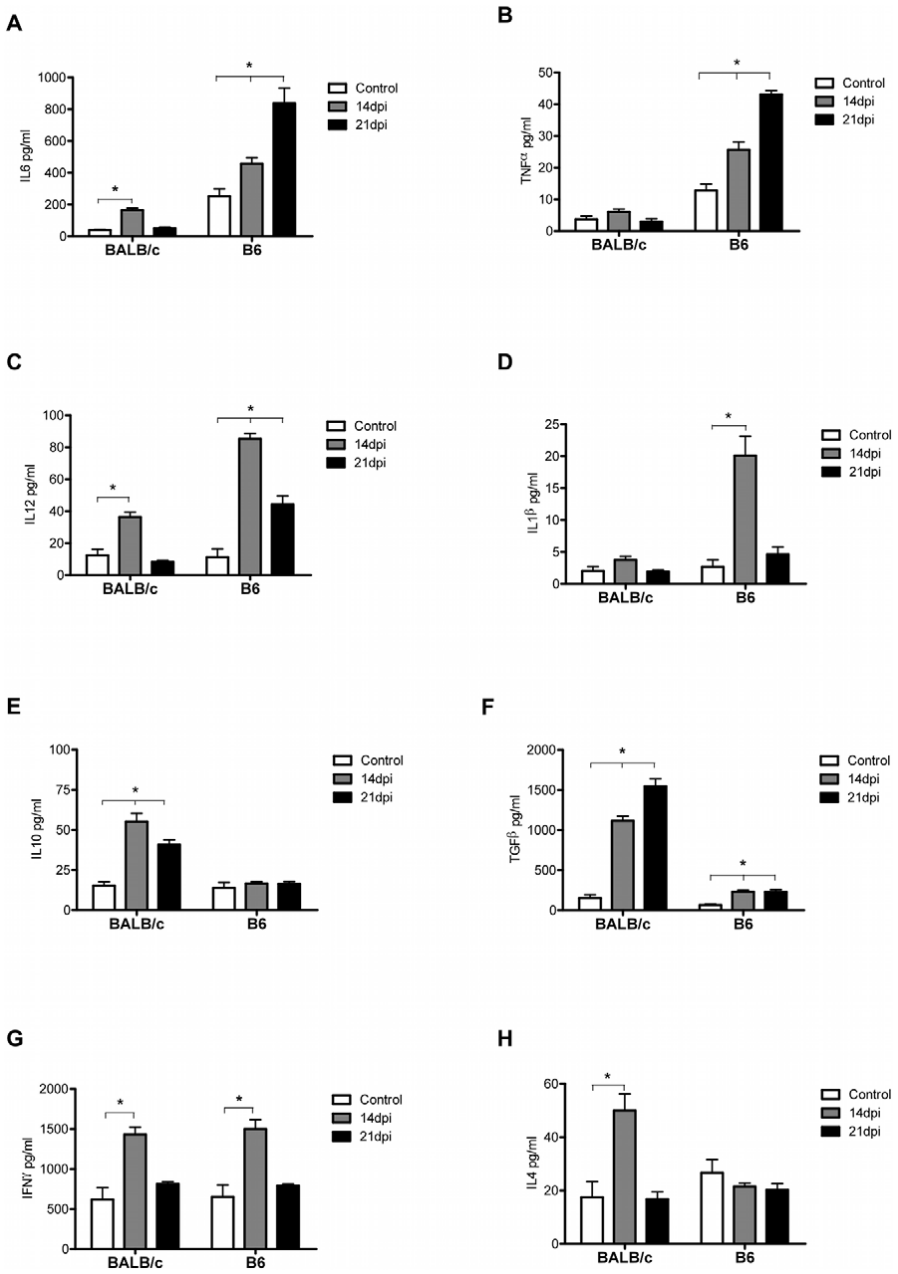
Cytokines produced by hepatic leukocytes from infected and control BALB/c and B6 mice. A–H) Purified leukocytes were cultured for 72 hs, and IL6, TNFα, IL12, IL1β, TGFβ, IL10, IFNγ and IL4 were assayed in supernatants. Six animals per group were analyzed and data are representative of one of three independent experiments. Statistical significance evaluated by Two-way ANOVA-Bonferroni's post-hoc test is indicated with a p-value <0.05. (*) To compare infected vs control mice.

The cytokine production was evaluated in supernatant of purified hepatic leukocytes by ELISA. A strong inflammatory profile, characterized by high levels of IL6, TNFα, IL12, and IL1β was found in B6 mice at 14 and 21 dpi compared to control mice (Figure 1A–D). BALB/c mice also showed an increase in IL6 and IL12 levels, but only at 14dpi (Figure 1A and C). In addition, TNFα and IL1β secretion by hepatic leukocytes from infected BALB/c did not produce any changes compared to non-infected mice (Figure 1B and D). The inflammatory response in liver was similar to our previously reported results using splenic leukocytes [13]. However and as opposed to spleen cells, purified liver leukocytes from B6 did not produce IL10 and only induced a slight increase in TGFβ (Figure 1E and F). Interestingly, hepatic leukocytes from infected BALB/c produced high levels of IL10 and TGFβ, two important immunoregulatory cytokines at 14 and 21dpi (Figure 1E and F). On analyzing the polarizing cytokines, we observed that *T. cruzi* infection induced a mixed Th1/Th2 profile with high levels of IFNγ and moderated production of IL4 in BALB/c, whereas high levels of IFNγ but not IL4 was detected in infected B6 mice (Figure 1G and H). These results indicate that *T. cruzi* acute infection induces a polarized Th1 profile in liver of B6 mice.

### High iNOS, gp91 and p47-phox NADPH oxidase expression in hepatic tissue and sustained NO production were detected in B6 liver leukocytes

**Figure 2:**
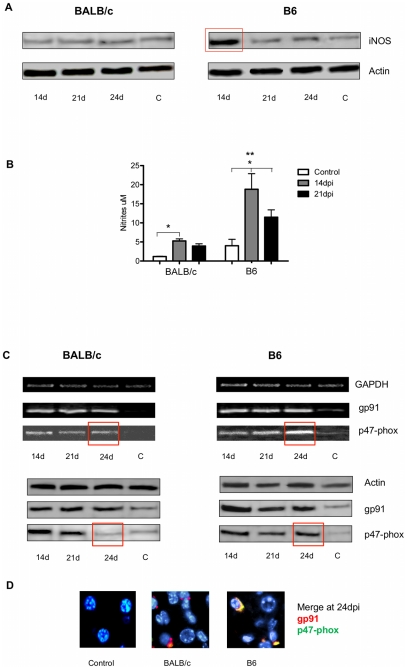
iNOS expression on hepatic tissue and nitrite production by liver leukocytes. Kinetics of gp91 and p47-phox NADPDH oxidase expression in liver tissues from infected and control BALB/c and B6 mice. A) Liver iNOS expression at14, 21 and 24 dpi, analyzed by Western blot. Non-infected control mice are indicated as “C”. The square indicates an iNOS significant increase in infected compared to control B6 mice. B) Nitrites were measured in supernatants of cultured hepatic leukocytes from infected (14 and 21dpi) and control mice. C) PCR products in agarose gel electrophoresis and western blot assays for GAPDH, actin, gp91 and p47-phox. The square indicates a significant increase of p47-phox subunit at 24dpi in B6 compared to BALB/c. D) Immunofluorescence merge for gp91 and p47-phox in control and infected liver from BALB/c and B6 mice, at 24dpi, are shown. Six animals/group were analyzed and data are representative of one of three independent experiments. A p-value <0.05 was considered significant using Two-way ANOVA test. (*) To compare infected vs control mice in each mouse strain. (**) To compare B6 vs BALB/c mice.

It is widely known that NO, which is produced by the enzyme iNOS, is a key mediator of a variety of biological functions, such as microbicidal activity and immunosuppression. However, NO is also associated with the most important immunopathologies, including septic shock [17], [18]. Taking into account that NO metabolite and Th1-biased response are involved in *T. cruzi* induced myocarditis [19] we quantified the iNOS induction and NO production as a possible mechanism involved in liver injury during this infection. As shown in Figure 2A, an increased iNOS expression was observed in hepatic tissue from B6 mice at 14dpi by Western blot. However, tissue from infected BALB/c did not reveal any changes in iNOS induction when compared to non-infected mice (Figure 2A). Nitrite production was increased in cultured hepatic leukocyte supernatants from both mouse strains at 14 dpi, but this was significantly lower in BALB/c than in B6. Additionally, B6 liver leukocytes showed a sustained nitrite production up to 21dpi (Figure 2B).

NADPH oxidase is a highly regulated membrane-bound enzyme complex found in a variety of phagocytic and non-phagocytic cells. The core enzyme consists of five subunits: p40-/p47-/p67-phox (cytosolic) and p22-/gp91-phox in plasma membrane. Upon stimulation, the cytosolic subunit p47phox is phosphorylated, and the entire cytosolic complex migrates to the membrane, where it associates with p-22-/gp91 to form active NADPH oxidase. Activation of NADPH oxidase leads to release of ROS to the extracellular environment, or at intracellular membranes. ROS production is important for killing pathogens, but an over production may be also detrimental to tissue homeostasis. In this work, we comparatively analyzed the induction of gp91 and p47-phox, two major components of the NADPH oxidase. The hepatic tissue from both mouse strains showed a significant increase in gp91 and p47-phox mRNA and proteins at 14, 21 and 24dpi compared to non-infected control (Figure 2C). Interestingly, p47-phox mRNA and protein expression was higher in B6 than in BALB/c at 24dpi (Figure 2C). Supporting this result, the activation of NADPH oxidase complex that involve co-localization in the plasma membrane of g91 and p47-phox subunits was observed only in infected B6 at 24dpi (Figure 2D).

### Different TLR2, TLR4 and TLR9 modulation in hepatic leukocytes and hepatocytes from BALB/c and B6 mice were induced

Normal and injured livers are enriched in innate immune cells, which have a significant impact on hepatic health or disease. Recently, we demonstrated that during *T. cruzi* acute infection the predominant leukocytes in liver were Gr1, F4/80 and CD3 positive cells [13]. These three populations accounted for more than 80% of the infiltrated liver cells from both infected BALB/c and B6 mouse strains.

**Figure 3:**
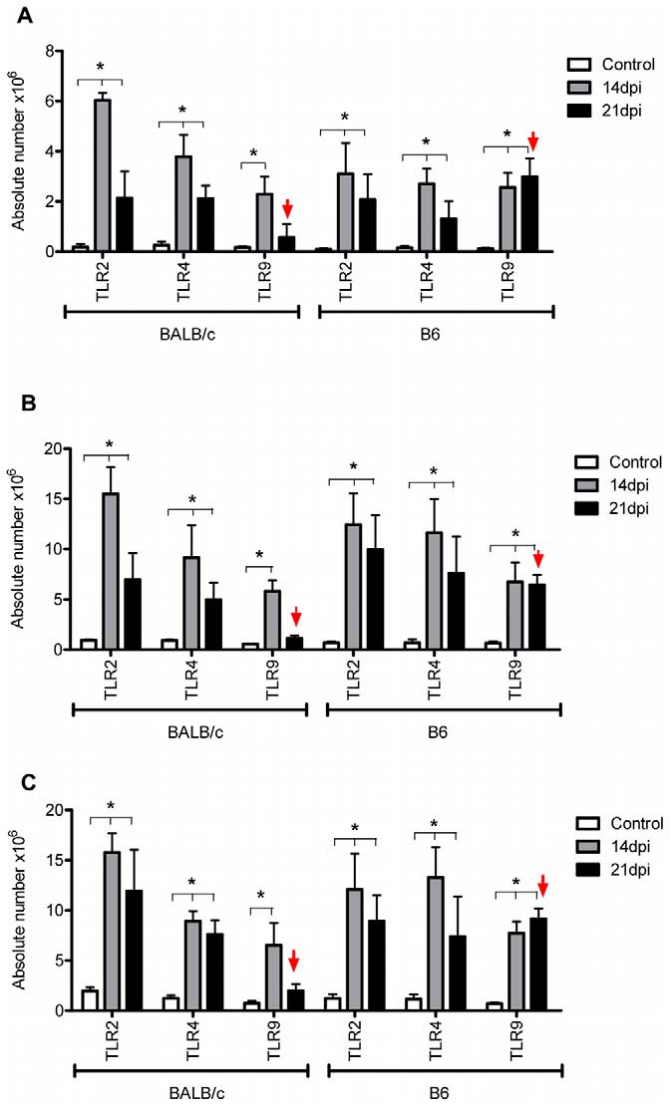
Positive CD3, Gr1 and F4/80 hepatic leukocytes expressing TLR2, TLR4 and TLR9. The TLR expression, after gating on CD3, Gr1 and F4/80, are showed in A, B and C respectively. Absolute numbers of double positive cells for CD3 (A), Gr1 (B) or F4/80 (C) and TLR2, TLR4 or TLR9 are shown. (*) Statistical significant increase comparing leukocytes from infected mice vs non-infected control mice in each mouse strain. Arrowhead indicates statistical difference in TLR9 expression at 21dpi comparing BALB/c vs B6. These assays were performed with six animals per group. Data are representative of one of three independent experiments. A p-value <0.05 was considered significant using Two-way ANOVA test.

**Figure 4:**
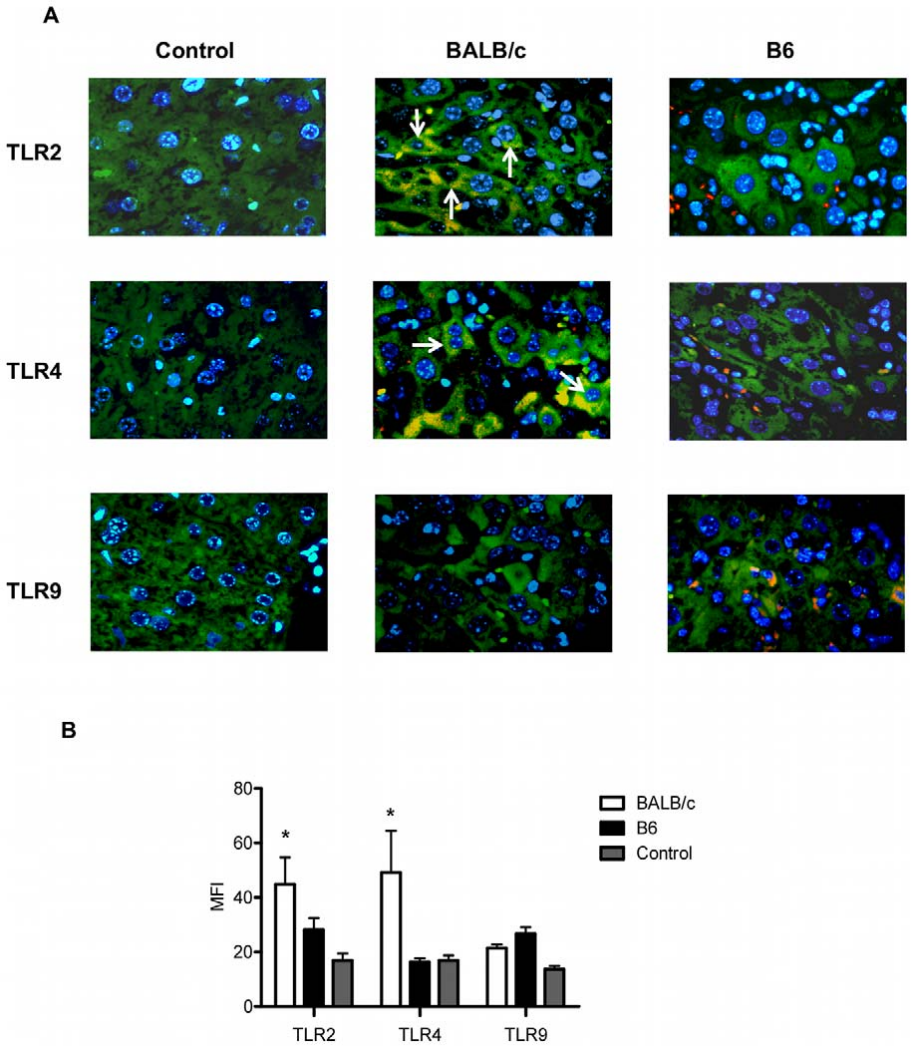
Cellular distribution of TLR2, TLR4 and TLR9 in hepatic tissue from infected (21 dpi) and control BALB/c and B6 mice. (A) Liver sections were co-stained with TLR2 or TLR4 or TLR9 (red) and cytokeratin (green) to show the co-localization between TLR/parenchyma cells. Nuclei were stained with Hoescht B) Intensity of TLR and cytokeratin merge was measured by LSM image examiner and representative values are shown. The bars represent the means and standard deviations of the co-localized mark intensity of 4 fields per group. The arrows indicate hepatocyte nuclei. Data are representative of one of three independent experiments. (*) Statistical significance compared to control levels is indicated (p-values <0,05).

In the present work, we analyzed the TLR2, TLR4 and TLR9 kinetic expression in CD3+ (Figure 3A), Gr1+ (Figure 3B) and F4/80+ (Figure 3C) hepatic leukocytes through double marking and employing flow cytometry. We observed that the absolute number for each double positive cell population expressing TLR2 and TLR4 was increased in both mouse strains at 14 and 21dpi compared to non-infected control mice. However, this increase did not show significant differences between both infected mouse strains (Figure 3A–C). Interestingly, double positive cells for TLR9 in CD3, Gr1 and F4/80 leukocytes were increased up to 21dpi only in infected B6 mice (Arrowhead in Figure 3A–C). Previously, we reported a decrease of TLR2 and TLR4 and an up-regulation of TLR9 in liver tissue of infected B6 mice, whereas BALB/c showed higher expression of TLR2 and TLR4 and a lower expression of TLR9 at the end of the acute phase [13]. Therefore, we think that other liver cells would be involved in the TLR2 and TLR4 differential expression observed between these mouse strains. Interestingly, the immunofluorescent analysis of TLR2, TLR4 and TLR9 expression showed higher TLR2 and TLR4 expression in hepatocytes from infected BALB/c compared to infected B6 mice (Figure 4A–B). These results clearly show a marked difference in the expression of TLR2 and TLR4 in hepatocytes from BALB/c and B6 during *T. cruzi* acute infection.

### Pre-treatment of B6 mice with TLR2 agonist before the infection reduced the local inflammation and liver damage during acute *T. cruzi* infection

**Figure 5:**
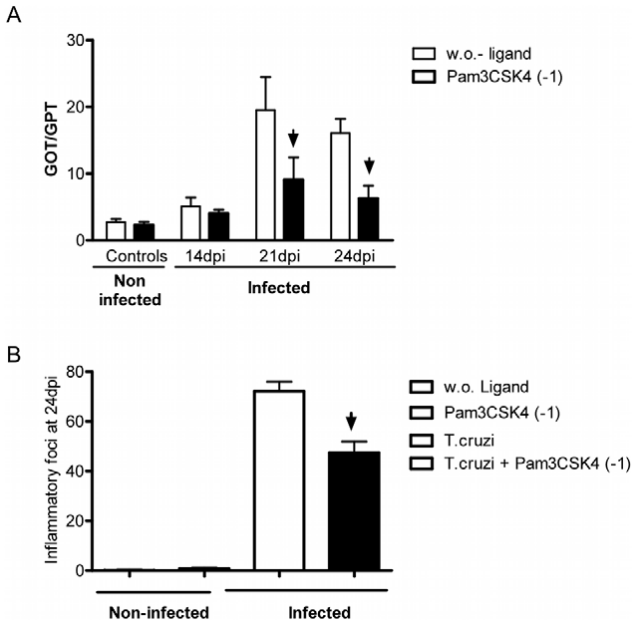
Plasma transaminase activity and inflammatory foci number in TLR-ligand pre-treated B6 mice. The mice were pre-stimulated 24hs before infection, with 10 ug of Pam3CSK4. A) Plasma samples from infected and non-infected mice were collected at the same time 14, 21 and 24dpi. GOT and GPT transaminase were measured at indicate times and GOT/GPT ratios are indicated. B) Inflammatory foci numbers were counted in 150 fields per liver section from different groups of treated B6 mice at 24 dpi. Average and standard deviation of four mice per group are shown. (*) Statistical significant (p-values <0,05 calculated by Two-way ANOVA test) are indicated and the results represent one of three independent experiments.

Taking into account that the TLR2 upregulation in hepatic tissue of infected BALB/c mice could be contributing to an anti-inflammatory effect and that TLR2 has been proposed as an immunomodulator receptor during *T.cruzi* infection [20], we evaluated whether pre-treatment with Pam3CSK4, a TLR2-TLR1 agonist, 24hs before infection with *T. cruzi* is able to improve the immune response balance and the outcome of the acute infection in B6 mice. Interestingly, the treatment of B6 mice with TLR2 agonist significantly reduced the GOT/GPT ratio compared to infected mice alone (Figure 5A). This result was very exciting because it indicates a marked diminution of liver damage during acute infection. Furthermore, the number of inflammatory foci (Figure 5B) and the mortality (Table 1) in mice pre-treated with TLR2 agonist were significantly lower than infected B6 mice and not treated mice. This result was correlated with lower transaminase activities (Pearson coefficient, r = 0.9) and consequently with a decreased tissue injury.

**Table 1:** Mortality percentage in pre-treated without infection, pre-treated and infected, and infected B6 mice.

	Pam3CSK4	Pam3CSK4+T. cruzi	T. cruzi
Mortality percentage at 24dpi	0	42,28+/−17.12	69,06+/−15,16

### Hepatic leukocytes from Pam3CSK4 pre-treated B6 mice showed less pro-inflammatory cytokine and NO production

**Figure 6:**
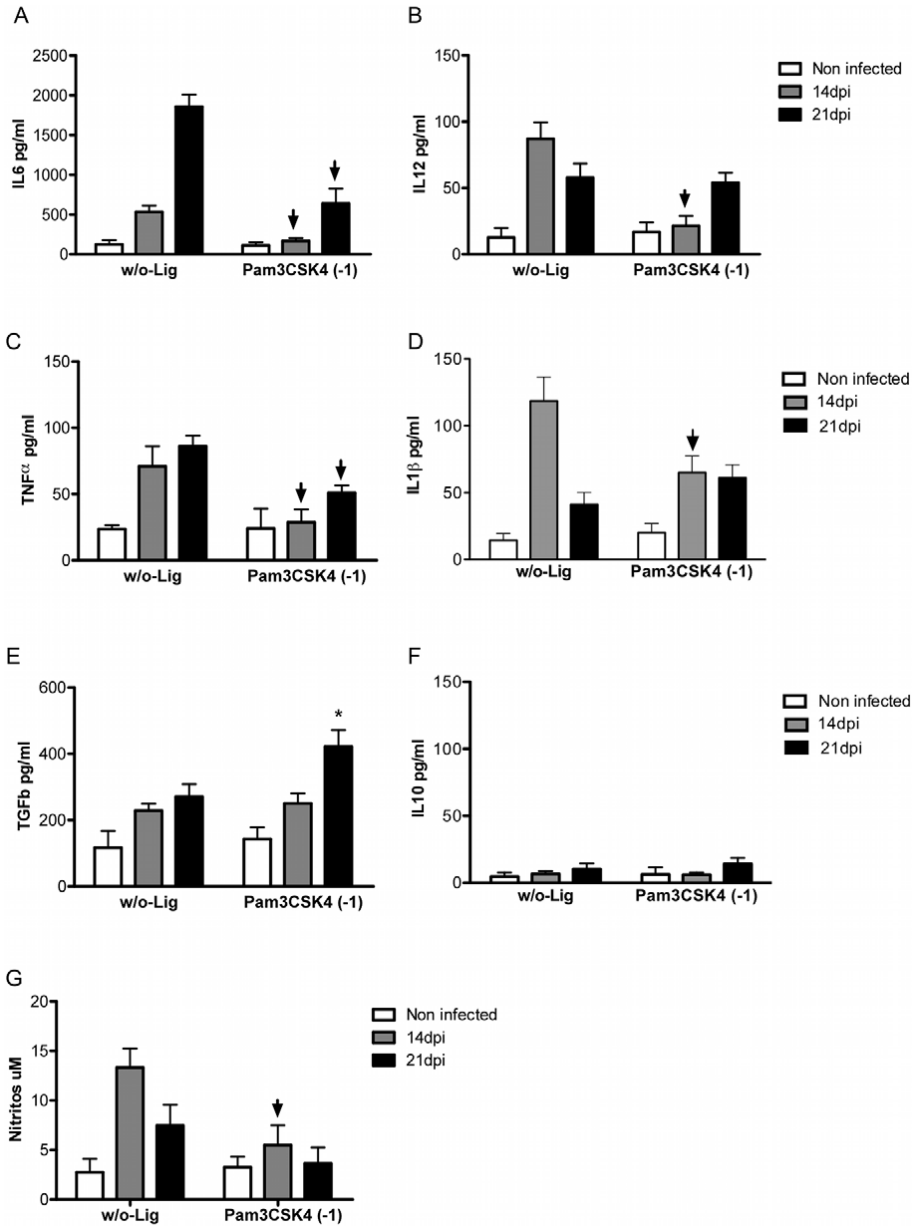
Cytokines and NO production by hepatic leukocytes from TLR-ligand pre-treated B6 mice. Purified liver leukocytes were obtained from each differently treated mice groups at 14 and 21dpi and cultured by 72hs. Supernatants were harvested and IL6 (A), IL12 (B), TNFα (C), IL1β (D), TGFβ (E) and IL10 (F) cytokines were determined by ELISA and NO (G) by Griess reaction. Four animals per group were analyzed and data are representative of one of three independent experiments. Arrowhead indicates statistical significant reduction (p<0,05) in pre-treatment infected mice compared to infected mice. (*) To indicate increase of TGFβ in pre-treated/infected vs infected B6 mice.

Testing the hypothesis that pre-treatment with Pam3CKS4 before *T. cruzi* infection would modulate the exacerbated inflammatory response in B6 mice, we demonstrate a reduction of IL6, IL12, TNFα and IL1β levels in pre-treated infected B6 mice compared to controls of infected mice alone (Figure 6A–D). In parallel, an increase of TGFβ concentration was detected in mice treated with TLR2 agonist (Figure 6E). Notably, NO secretion was also reduced in mice pre-treated compared to infected mice alone at 14dpi (Figure 6G).

Our results suggest that the pre- treatment with Pam3CSK4 one day before infection reduces the inflammatory environment and the hepatic damage of B6 mice during *T. cruzi* infection.

## Discussion

In the present work, we focus on the study of local immune response in hepatic tissue during *T. cruzi* infection. Our results clearly demonstrate that liver leukocytes from infected B6 mice produce increased and persistent levels of TNFα, IL6, IL12 and IL1β pro-inflammatory cytokines, associated with undetectable or low IL10 and TGFβ production. Previously, we observed a enhanced apoptosis and necrosis in liver of this mouse strain [13]. Recently, it was reported that anti-TNFα treatment decreases hepatic apoptosis of C57BL/6 mice infected with trypomastigotes of Tulahuén strain, providing evidence about the role of this cytokine in the induction of liver injury [12]. By contrast, in livers from BALB/c mice, the pro-inflammatory profile was effectively countered by IL10 and TGFβ regulatory cytokines. In this mouse strain, infection induced a mixed Th1/Th2 phenotype while a dominant Th1 profile was observed in livers of infected B6 mice. The Th1 response contributes to an effective parasite clearance although an excessive inflammation may be deleterious for the host. We postulated that a mixed cytokine prototype in BALB/c might be insufficient for controlling parasite but might favour tissue repair. Similarly, a mixed Th1/Th2 and a dominant Th1 phenotype was observed in heart of infected BALB/c and B6 mice respectively [21].

Nitric oxide and ROS are two key inflammatory mediators involved not only in pathogen clearance but also in tissue injury. Nitric oxide is produced by different isoforms of NO synthase, among them the inducible isoform (iNOS) that is activated by IFNγ and TNFα [22], two cytokines that were prominent in our model. Hemodynamic instabilities and tissue damage caused by NO may be related to different NO synthase isoforms [23] as well as to differences in cellular concentration and/or temporal expression [17], [24]. In our work, we demonstrated a stronger expression of hepatic iNOS and a higher NO production by liver leukocytes of infected B6 compared to BALB/c mice. Interestingly, this process was concomitant with high levels of pro-inflammatory cytokines detected in B6 mice.

On the other hand, results presented here demonstrate that p47 and gp91-phox components of NADPH oxidase were increased in livers of both infected mouse strains compared to non-infected. However, an enhanced and sustained p47-phox expression was observed in livers from B6 mice. Strikingly, the co-expression of gp91 and p47-phox on the plasma membrane was found only in liver from infected B6 at 24dpi. Several authors have described that ROS can induce cell death by either apoptosis or necrosis in liver pathologies [25], [26]. Thus, the activation of NADPH oxidase enzymatic complex would be a key player in the liver damage, probably as an instrument contributing to liver apoptosis and necrosis during infection in B6 mice.

TLRs have been shown to be critical for the protection against infections. Recently, Padilla *et al*. 2009, demonstrated that insufficient TLR activation contributes to the slow development of CD8+ T cell responses in *T. cruzi* infection [16]. However, the breakdown of homeostasis by infection can deregulate certain pathways, such as TLR-cytokines, and plays a key role in the pathogenesis of the liver disease. Our results show a similar TLR2 and TLR4 expression on hepatic macrophages, granulocytes and T cells of infected BALB/c and B6 mice. However, the TLR9 expression showed a clear difference in hepatic leukocytes from infected B6 and BALB/c mice. We found that only leukocytes from infected B6 mice maintained high expression of TLR9 during the acute phase. These results support the hypothesis that sustained TLR9 signalling might contribute to excessive and harmful inflammatory response in infected B6 mice. In accordance with our results, a crucial role of TLR9 in IL12 and IFNγ production during *T. cruzi* infection was recently shown [15],[27]. Furthermore, it is noteworthy that uninfected BALB/c and B6 mice did not show differences in their TLR9 expression, yet the persistent levels of this receptor in infected B6 could involve some lost factors, that are present in BALB/c, which should modulate the excessive inflammatory response in liver. Interestingly, we found that TLR2 and TLR4 are differentially modulated in hepatocytes of infected BALB/c and B6 mice, suggesting that these innate immune receptors would play a role not only in immune cells but also in liver parenchyma cells. In this sense, it has been postulated that TLR signalling in parenchyma cells would be a key mechanism to prevent death caused by excessive cytokine release [28]–[30]. There are increased evidences demonstrating the potential role of TLR-ligands treatment as therapeutic approach and they have shown to be highly effective in the protection against protozoan, among them *T. cruzi* [15], [31], [32]. In our study we observed that pre-treatment with Pam3CSK4, a TLR2-TLR1 agonist, before infection induced a marked reduction of pro-inflammatory cytokines, nitrite and transaminasa levels and a decrease in the number of hepatic inflammatory foci and consequently in the mortality of infected mice.

Together these results suggest that TLRs and probably others PRR should contribute to the induction of anti-parasite immune response, but an altered TLR regulation could lead to tissue damage. Interestingly, TLRs sense the endogenous molecules released by dying cells and lead to fast IL1β release through inflammasome activation [33]. Notably, in our model the IL-1β cytokine was up regulated in infected B6 mice and it would be a very interesting idea to explore activation of inflammasome platform during acute phase of *T. cruzi* infection.

In our study we postulated that the inadequate integration of signals involving molecular (TLRs, cytokines, NO and ROS) and cellular (immune and parenchyma cells) components influences the outcome of local immune response during this parasite infection. Moreover, the differential TLR and cytokine modulation in the liver, induced by *T. cruzi* infection, emphasize the importance of local innate immune response in hosts with different genetic background and could contribute to the understanding and design of novel immune strategies in controlling liver pathologies.
