# Correction: Importance of TLR2 on Hepatic Immune and Non-Immune Cells to Attenuate the Strong Inflammatory Liver Response During *Trypanosoma cruzi* Acute Infection

**DOI:** 10.1371/journal.pntd.0011738

**Published:** 2023-11-01

**Authors:** Eugenio Antonio Carrera-Silva, Natalia Guiñazu, Andrea Pellegrini, Roxana Carolina Cano, Alfredo Arocena, Maria Pilar Aoki, Susana Gea

After this article [1] was published, concerns were raised about some of the PCR gels in Figure 2. Specifically, in Figure 2C, the B6 GAPDH panel is duplicated as the BALB/c GAPDH panel.

**Figure 2:**
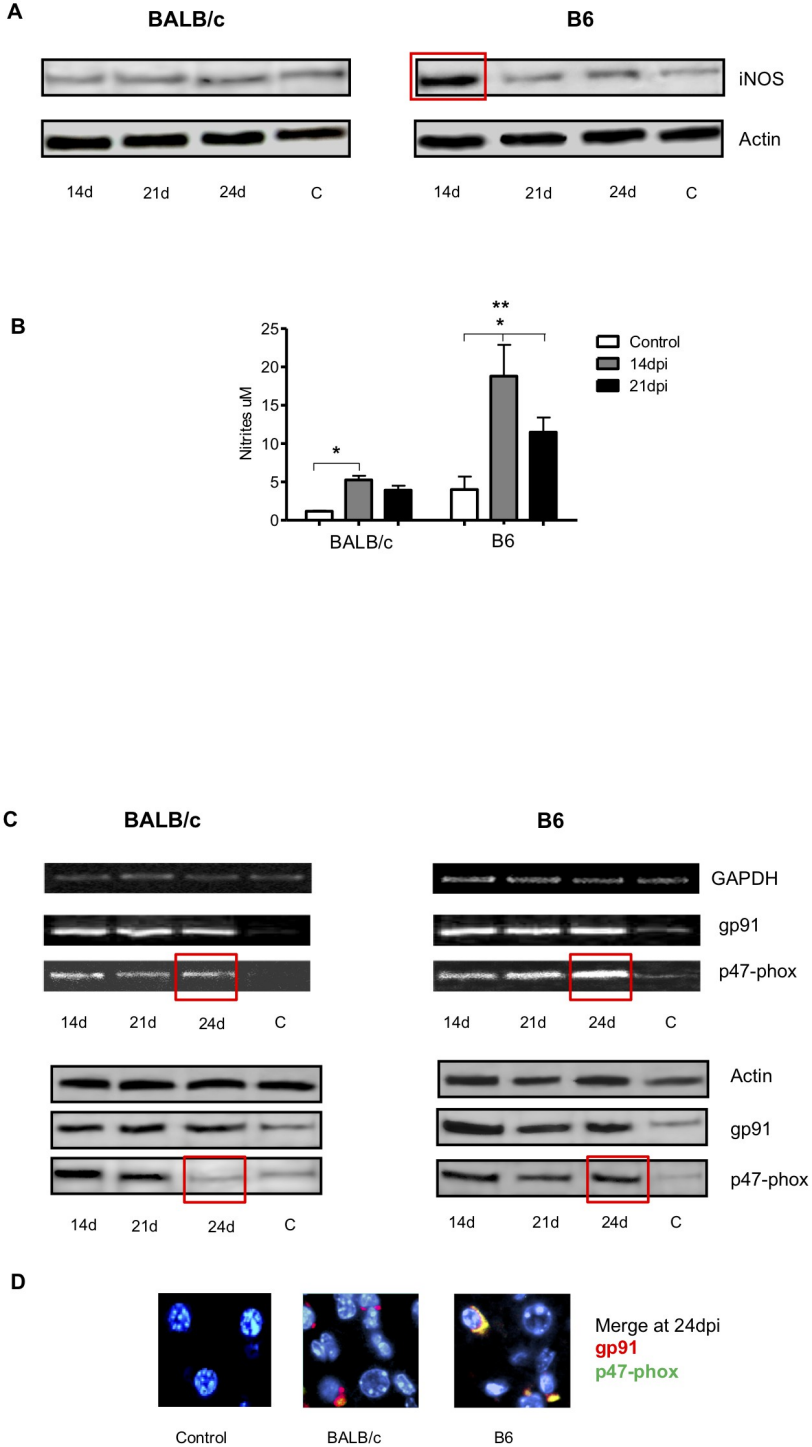
iNOS expression on hepatic tissue and nitrite production by liver leukocytes. Kinetics of gp91 and p47-phox NADPDH oxidase expression in liver tissues from infected and control BALB/c and B6 mice. A) Liver iNOS expression at14, 21 and 24 dpi, analyzed by Western blot. Noninfected control mice are indicated as ‘‘C”. The square indicates an iNOS significant increase in infected compared to control B6 mice. B) Nitrites were measured in supernatants of cultured hepatic leukocytes from infected (14 and 21dpi) and control mice. C) PCR products in agarose gel electrophoresis and western blot assays for GAPDH, actin, gp91 and p47-phox. The square indicates a significant increase of p47-phox subunit at 24dpi in B6 compared to BALB/c. D) Immunofluorescence merge for gp91 and p47-phox in control and infected liver from BALB/c and B6 mice, at 24dpi, are shown. Six animals/group were analyzed and data are representative of one of three independent experiments. A p-value <0.05 was considered significant using Two-way ANOVA test. (*) To compare infected vs control mice in each mouse strain. (**) To compare B6 vs BALB/c mice.

In response to queries about the experiments in Figure 2C, the first author stated that the B6 GAPDH panel was duplicated as the BALB/c GAPDH panel in error. An updated version of Figure 2 with the correct BALB/c GAPDH housekeeping gen panel in Figure 2C from the time of the original experiments is provided here. The original image data underlying the B6 GAPDH and BALB/c GAPDH panels in Figure 2C are provided here in S1 File.

The remaining analyzed and processed data, but not some of the raw unprocessed files, and all individual-level quantitative data underlying article [1] are available from the first author.

The authors apologize for the error in the published article.

## Supporting information

S1 File Original underlying images for the B6 GAPDH and BALB/c GAPDH panels in Figure 2C. (PPTX)
