# Supplementary figures and images for: Correction: Importance of TLR2 on Hepatic Immune and Non-Immune Cells to Attenuate the Strong Inflammatory Liver Response During Trypanosoma cruzi Acute Infection
Source: PLoS Negl Trop Dis. 2023 Nov 1;17(11):e0011738. doi: 10.1371/journal.pntd.0011738 (PMC10619868; doi:10.1371/journal.pntd.0011738)

## Slide 1
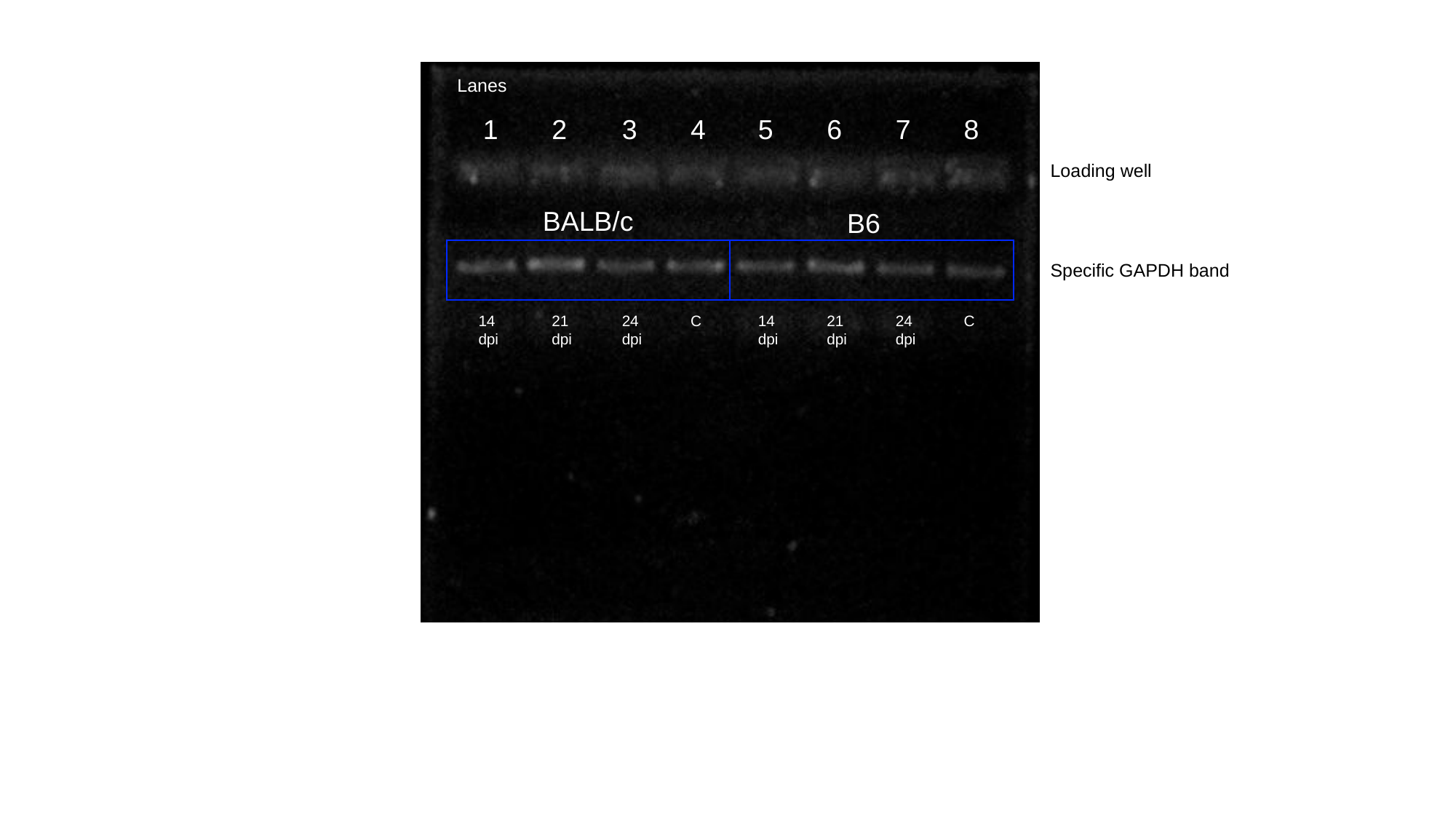

Lanes
1
2
3
4
5
6
7
8
Loading well
BALB/c
B6
Specific GAPDH band
14dpi
21 dpi
24dpi
C
14 dpi
21dpi
24dpi
C

Supplement: S1 File — (PPTX) [file pntd.0011738.s001.pptx]
